# Supplementary material for: New Epoxy Thermosets Organic-Inorganic Hybrid Nanomaterials Derived from Imidazolium Ionic Liquid Monomers and POSS®Ph
Source: Nanomaterials (Basel). 2022 Feb 6;12(3):550. doi: 10.3390/nano12030550 (PMC8837961; doi:10.3390/nano12030550)
Supplement: Supplementary file 1 [file nanomaterials-12-00550-s001.zip › nanomaterials-1550061-supplementary.pdf]

nanomaterials

Supplementary Materials

New Epoxy Thermosets Organic-Inorganic Hybrid Nanomaterials Derived from Imidazolium Ionic Liquid Monomers and POSS$^{®Ph}$

Houssém Chabane [1,2], Sébastien Livi [1], Jannick Duchet-Rumeau [1] and Jean-François Gérard [1,*]

1 Univ Lyon, CNRS, UMR 5223, Ingénierie des Matériaux Polymères, Université Claude Bernard Lyon 1, INSA Lyon, Université Jean Monnet, F-69621 France Cédex, France; houssem.chabane@insa-lyon.fr (H.C.); sebastien.livi@insa-lyon.fr (S.L.); jannick.duchet@insa-lyon.fr (J.D.-R.)
2 Laboratoire de Chimie Macromoléculaire, Ecole Militaire Polytechnique, BP 17, Bordj El-Bahri, 16111 Algiers, Algeria
* Correspondence: jean-francois.gerard@insa-lyon.fr; Tel.: + 33 04 72 43 60 04 (J.F.G.); + 33 06 05 52 32 29 (H.C.)

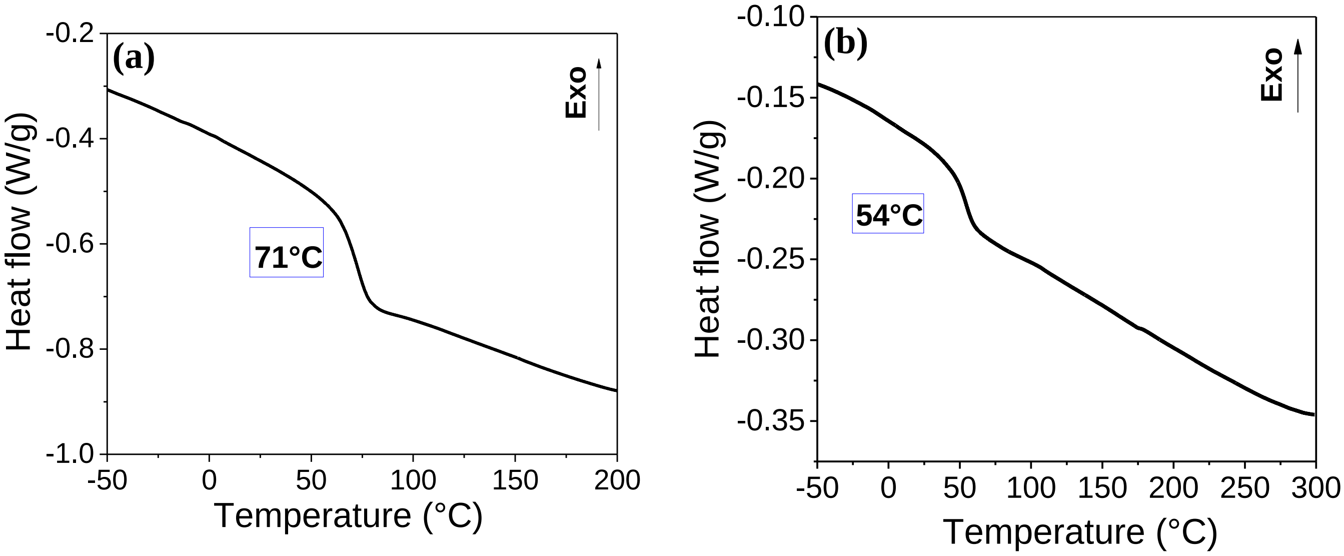

Figure S1. DSC thermograms after curing of (**a**) ILM1/IPD and (**b**) ILM2/IPD.

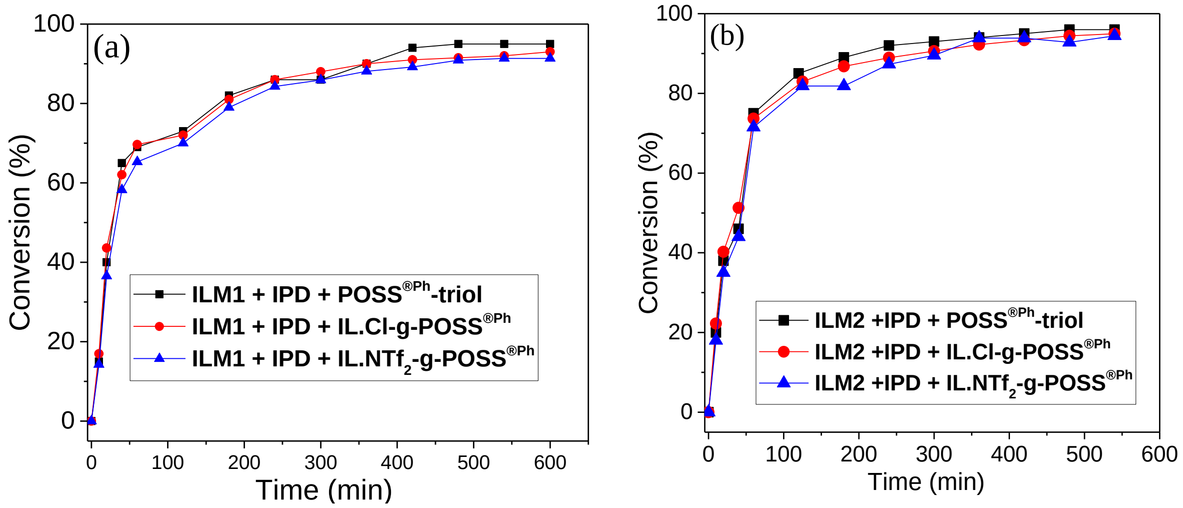

Figure S2. Epoxy group conversion as a function of the reaction time and during temperature steps from FT-IR for the epoxy O/I hybrid networks containing POSS$^{®Ph}$-triol, IL.Cl-g-POSS$^{®Ph}$ and IL.NTf$_2$-g-POSS$^{®Ph}$ respectively, prepared based on epoxy ionic liquids monomers: (**a**) ILM1, and (**b**) ILM2.

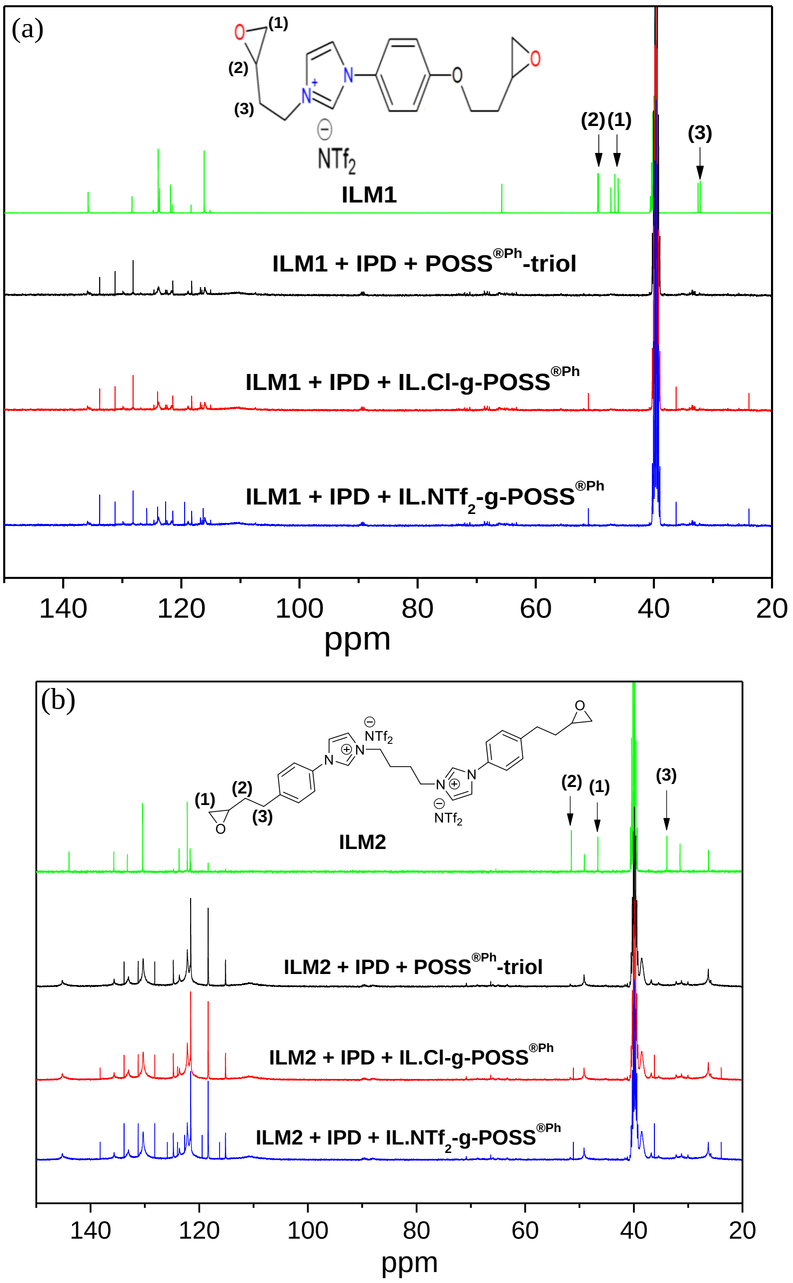

Figure S3. HR-MAS ^{13}C-NMR spectrum of the epoxy O/I hybrid networks containing POSS$^{®Ph}$-triol, IL.Cl-g-POSS$^{®Ph}$ and IL.NTf$_2$-g-POSS$^{®Ph}$ respectively, prepared based on epoxy ionic liquids monomers: (**a**) ILM1, and (**b**) ILM2 (DMSO-d$_6$; 400 MHz).

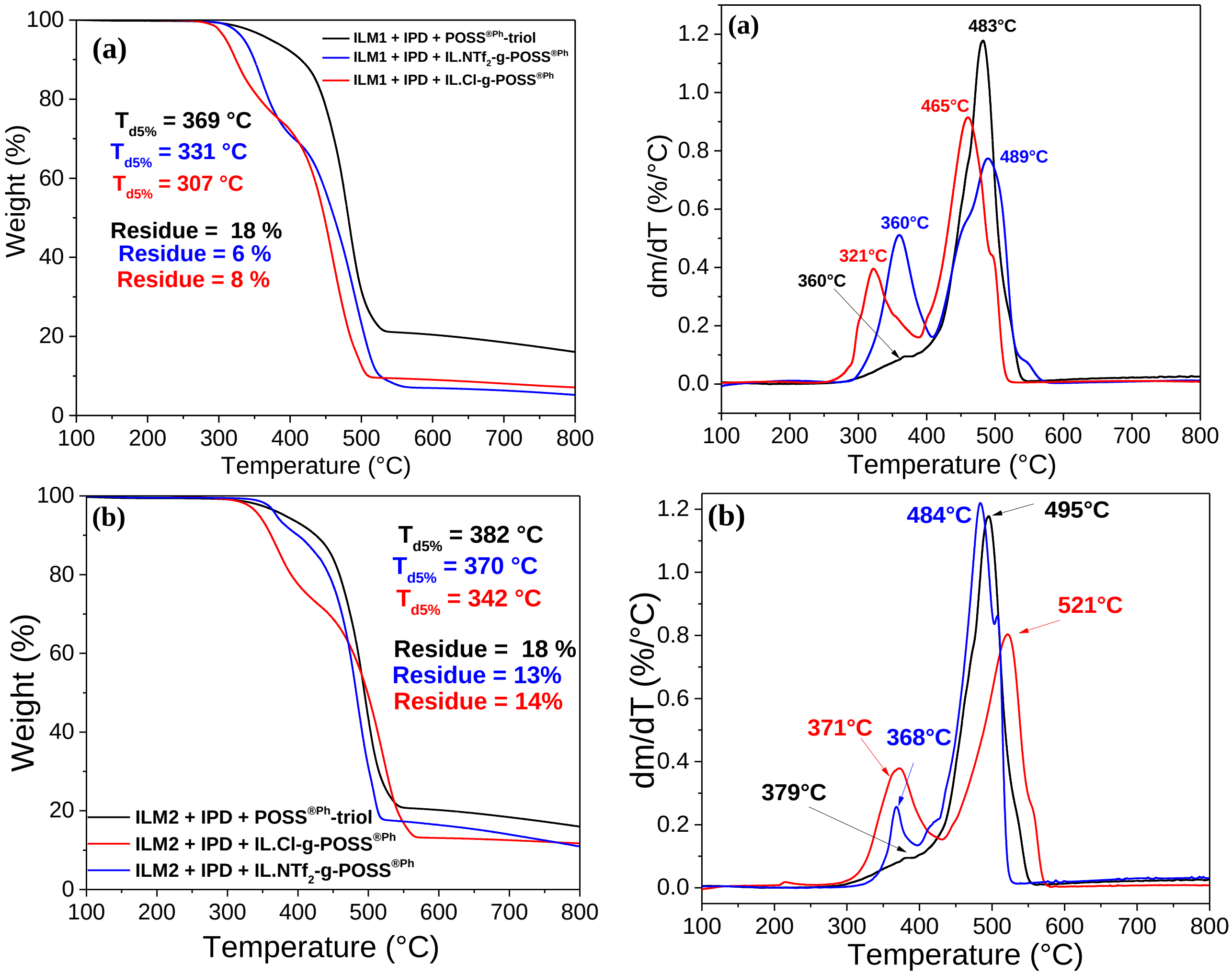

Figure S4. Evolution of weight loss as a function of the temperature (TGA) and derivative of TGA curves (DTG) for the epoxy hybrid O/I networks containing POSS®Ph-triol, IL.Cl-g-POSS®Ph and IL.NTf2-g-POSS®Ph respectively, prepared based on epoxy monomers: (**a**) ILM1, and (**b**) ILM2.

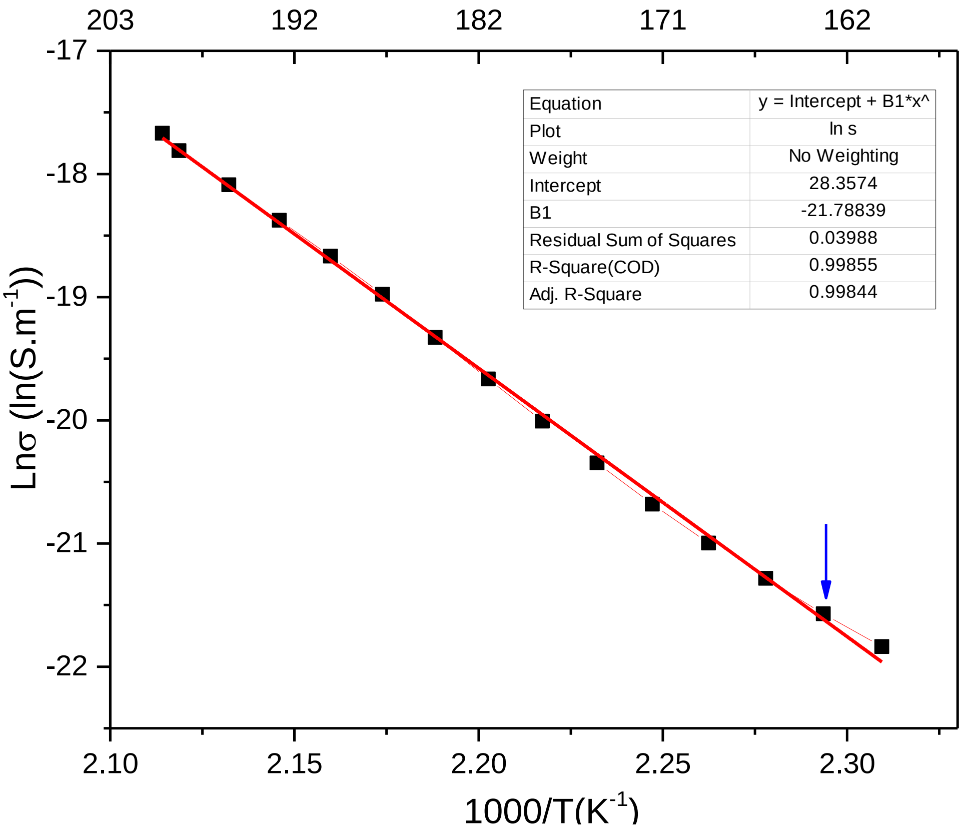

Figure S5. Dependence of DC conductivity with temperature (extrapolated from AC conductivity values at 0.1 Hz for DGEBA/IPD network). Solid red line represents a regression to the Arrhenius equation.
